# Transcriptional Control of Steroid Biosynthesis Genes in the *Drosophila* Prothoracic Gland by Ventral Veins Lacking and Knirps

**DOI:** 10.1371/journal.pgen.1004343

**Published:** 2014-06-19

**Authors:** E. Thomas Danielsen, Morten E. Moeller, Elad Dorry, Tatsuya Komura-Kawa, Yoshinori Fujimoto, Jesper T. Troelsen, Rachel Herder, Michael B. O'Connor, Ryusuke Niwa, Kim F. Rewitz

**Affiliations:** 1 Department of Biology, University of Copenhagen, Copenhagen, Denmark; 2 Graduate School of Life and Environmental Sciences, University of Tsukuba, Tsukuba, Ibaraki, Japan; 3 Department of Chemistry and Materials Science, Tokyo Institute of Technology, Meguro, Tokyo, Japan; 4 Department of Science, Systems and Models, Roskilde University, Roskilde, Denmark; 5 Department of Genetics, Cell Biology and Development, University of Minnesota, Minneapolis, Minnesota, United States of America; 6 PRESTO, JST, Kawaguchi, Saitama, Japan; K.U.Leuven, Belgium

## Abstract

Specialized endocrine cells produce and release steroid hormones that govern development, metabolism and reproduction. In order to synthesize steroids, all the genes in the biosynthetic pathway must be coordinately turned on in steroidogenic cells. In *Drosophila*, the steroid producing endocrine cells are located in the prothoracic gland (PG) that releases the steroid hormone ecdysone. The transcriptional regulatory network that specifies the unique PG specific expression pattern of the ecdysone biosynthetic genes remains unknown. Here, we show that two transcription factors, the POU-domain Ventral veins lacking (Vvl) and the nuclear receptor Knirps (Kni), have essential roles in the PG during larval development. Vvl is highly expressed in the PG during embryogenesis and is enriched in the gland during larval development, suggesting that Vvl might function as a master transcriptional regulator in this tissue. Vvl and Kni bind to PG specific cis-regulatory elements that are required for expression of the ecdysone biosynthetic genes. Knock down of either *vvl* or *kni* in the PG results in a larval developmental arrest due to failure in ecdysone production. Furthermore, Vvl and Kni are also required for maintenance of TOR/S6K and prothoracicotropic hormone (PTTH) signaling in the PG, two major pathways that control ecdysone biosynthesis and PG cell growth. We also show that the transcriptional regulator, Molting defective (Mld), controls early biosynthetic pathway steps. Our data show that Vvl and Kni directly regulate ecdysone biosynthesis by transcriptional control of biosynthetic gene expression and indirectly by affecting PTTH and TOR/S6K signaling. This provides new insight into the regulatory network of transcription factors involved in the coordinated regulation of steroidogenic cell specific transcription, and identifies a new function of Vvl and Knirps in endocrine cells during post-embryonic development.

## Author Summary

Steroid hormones play important roles in physiology and disease. These hormones are molecules produced and secreted by endocrine cells in the body and control sexual maturation, metabolism and reproduction. We found transcriptional regulators that underlie the specialized function of endocrine steroid-producing cells. In the steroid-producing cells of the fruit fly *Drosophila*, Ventral veins lacking (Vvl) and Knirps (Kni) turn on all the genes required for steroid production. When Vvl or Kni were inactivated in the cells where the hormone is made, the genes involved in steroid production were not activated. Because of the reduced steroid production, the juvenile larvae failed to develop and undergo maturation to adulthood. Inactivation of Vvl and Kni also reduces endocrine cell growth by disturbing their response to growth promoting signals. Genetic variations in humans with the loss of a homolog of Vvl have been associated with disorders caused by insufficient steroid production. Together with the fact that Vvl is highly expressed in the steroid-producing cells of *Drosophila*, this suggests that Vvl may be a conserved master regulator of steroid production. Our findings provide insight into the network of factors that control endocrine cell function and steroid hormone levels that could have implication for human diseases.

## Introduction

Steroid hormones have a conserved role in the regulation of developmental transitions, growth, metabolism and reproduction in animals [1]-[3]. Specialized endocrine tissues with cell-type specific complements of enzymes that form biochemical pathways mediate the biosynthesis of steroids. In *Drosophila* larvae, the steroid biosynthetic enzymes are expressed in the prothoracic gland (PG), the endocrine tissue of insects and the major source of the steroid hormone ecdysone. The production of ecdysone in the PG is regulated by a checkpoint control system in response to external and internal signals [2]. These checkpoints allow the endocrine system to assess growth and nutrient status before activating the biochemical pathway that increases the release of ecdysone, which triggers developmental progression.

Despite the importance of the coordinated expression in endocrine cells of the steroidogenic enzymes, the PG specific transcriptional regulatory networks that underlie steroidogenic cell function remain unknown. The steroidogenic function of the PG cells is defined by the restricted expression of the genes involved in ecdysone biosynthesis that mediate the conversion of cholesterol to ecdysone. The components of the ecdysone biosynthetic pathway include the Rieske-domain protein Neverland (Nvd) [4], [5], the short-chain dehydrogenase/reductase Shroud (Sro) [6] and the P450 enzymes Spook (Spo), Spookier (Spok), Phantom (Phm), Disembodied (Dib) and Shadow (Sad) [7]–[12] collectively referred to as the Halloween genes. Ecdysone produced by the PG is released into circulation and converted into the more active hormone, 20-hydroxyecdysone (20E), in peripheral tissues by the P450 enzyme, Shade (Shd) [13], [14].

The cell-type specific pattern and precise dynamics of the ecdysone titers suggest a tight transcriptional regulation of the biosynthetic enzymes in the PG. This is likely orchestrated by multiple transcription factors working in a network to achieve spatial and temporal control of steroid hormone production during development. The composition of this tissue-specific transcriptional regulation remains largely elusive, although some transcription factors are known to regulate ecdysone production in the PG [15]-[18]. The nuclear receptor DHR4 functions as a repressor of ecdysone biosynthesis in the PG and responds to prothoracicotropic hormone (PTTH) mediated activation of the mitogen-activated protein kinase (MAPK) pathway [17]. Loss of *βFTZ-F1* in the PG has also been associated with reduced expression of *phm* and *dib* [18]. The zing-finger protein Without children (Woc) is required for ecdysone biosynthesis [19], although the pathway component regulated by Woc has not been identified. However, it is unclear if Woc, βFTZ-F1 and DHR4 bind directly to the regulatory regions that control expression of the ecdysone biosynthetic genes. In contrast, we recently showed that the transcription factor Broad (Br) regulates expression of the genes involved in ecdysone biosynthesis by direct binding to their promoters/enhancers [20]. Although these factors may be important for steroidogenic gene expression, other factors are likely required for the transcriptional regulatory network that defines the PG cell-specific expression of the ecdysone biosynthetic pathway components.

We recently characterized cis-regulatory elements required for the expression of *phm* and *dib* in the *Drosophila* PG [20], including a 69 bp promoter element located in the upstream *phm* region and a 86 bp region in the third intron of *dib*. These elements are important for the temporal up-regulation of *phm* and *dib* by Br isoform 4 (Br-Z4) that increases the ecdysteroidogenic capacity of the PG and allows the production of the high-level ecdysone pulse that triggers pupariation. To further characterize the tissue-specific regulation of the ecdysone biosynthetic pathway, we analyzed PG specific regulatory elements for the presence of transcription factor binding sites.

Here, we report a novel role for Ventral veins lacking (Vvl) and Knirps (Kni) in regulating ecdysteroidogenesis in *Drosophila*. The cis-regulatory elements responsible for PG specific expression of *spok*, *phm* and *dib* contain conserved Vvl and Kni binding sites. Expression of *vvl* is high in the PG compared to the whole animal, while *kni* expression is less PG-specific. Knock down of *vvl* and *kni* in the PG results in larval developmental arrest due to impaired ecdysone production. We show that Vvl and Kni specifically regulate expression of all the ecdysone biosynthetic enzymes through functionally important regulatory sites. Furthermore, we find that Molting defective (Mld) specifically regulates enzymes that catalyze early steps in the ecdysone biosynthetic pathway. Our study identifies Vvl as a PG cell-specific transcription factor that underlies steroidogenic cell function. We conclude that Vvl and Kni are involved in the transcriptional regulatory network of the PG that coordinates expression of biosynthetic enzymes required for ecdysone production during *Drosophila* development.

## Results

### Regulatory regions of ecdysone biosynthesis genes contain conserved binding sites for Vvl and Kni

**Figure 1:**
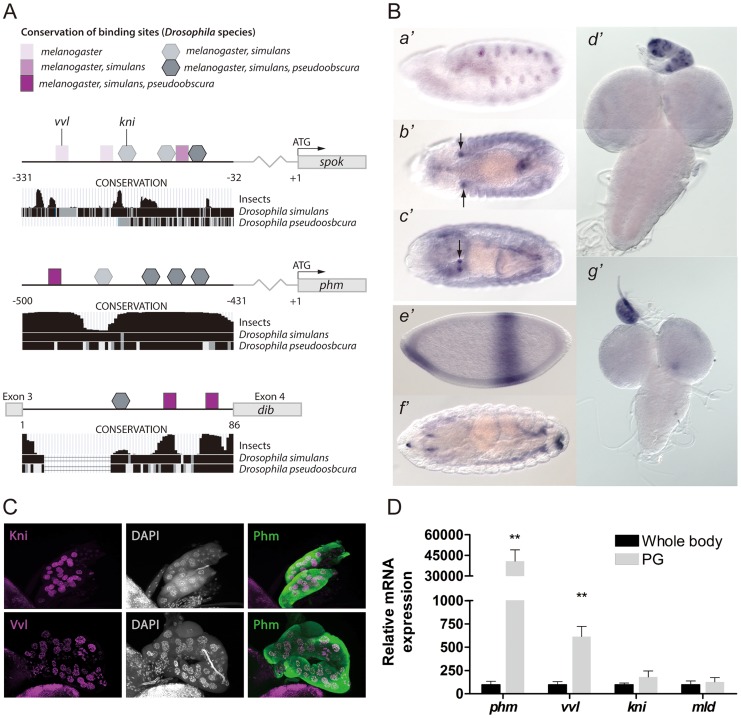
Vvl and Kni have binding sites in the promoters and enhancers of the ecdysone biosynthetic genes and are expressed in the PG. (A) An illustration showing binding sites in the PG specific cis-regulatory elements of *spok* and *phm* and *dib*. Binding sites are indicated by squares (Vvl) and pentagons (Kni) with shades indicating the conservation of the site between *Drosophila* species. Conservation tracks were obtained from the UCSC genome browser. (B) *In situ* hybridization of embryos and third instar larval brains and ring glands with antisense probes for *vvl* (*a*′–*d*′) or *kni* (*e*′–*g*′). (*a*′) Stage 11, shows *vvl* expression in the primordial cells of the trachea, while (*b*′) stage 13, (*c*′) stage 16 and (*d*′) L3 show strong *vvl* expression in the PG cells of the ring gland. (*e*′*)* stage 4, (*f*′*)* stage 16 and (*g*′) L3 show *kni* expression in the PG of L3 larvae, but not clearly in embryos. (C) Immunostaining of the PG from L3 larvae with antibodies against Kni and Vvl (magenta) and Phm (green). Co-localization with nuclei staining (DAPI: gray) indicates that Vvl and Kni are expressed in the nucleus of the PG cells. (D) Expression of *phm*, *vvl*, *kni* and *mld* measured by qPCR in tissue from whole body L3 larvae or dissected ring glands containing the PG of L3 larvae (n = 4). *vvl* is highly expressed in the ring gland compared to whole body, like *phm*, while the expression of *kni* and *mld* show a minor enrichment in the gland. Error bars indicate s.e.m. **P<0.01, versus whole body.

We analyzed the *phm* and *dib* PG specific regulatory elements for transcription factor binding sites. Our *in silico* search revealed conserved binding sites for the POU-domain transcription factor Vvl and the nuclear receptor Kni in the *phm* promoter and *dib* enhancer (Fig. 1A and S1). Analysis of the *phm* promoter identified one conserved Vvl site and four Kni sites of which three are highly conserved, indicating that they are important regulatory sites. In support of this, mutations disrupting the Vvl site and one of the conserved Kni sites eliminate PG specific GFP reporter expression [20]. In contrast, mutations in the non-conserved Kni binding sites do not reduce PG expression. The third intron *dib* enhancer also contains one Vvl site and two Kni sites, both in regions that have been conserved. We also identified a 300 bp PG specific promoter for *spok*, encoding an enzyme that acts at an early step in the ecdysone biosynthetic pathway [9]. This element located −331 to −32 bp upstream of translation start drives specific PG reporter GFP expression. This *spok* promoter contains three Vvl and three Kni binding sites, although these sites are less conserved compared to the Vvl and Kni sites identified in the *phm* and *dib* regulatory elements. Expression of *spok* has previously been reported to require Molting defective (Mld), a nuclear zinc finger protein [9]. Since the DNA binding sequence motif for Mld has not yet been characterized, we were unable to examine potential Mld binding sites in the *spok* promoter.

### Vvl and Kni are expressed in the prothoracic gland

The observation of Vvl and Kni binding sites in the promoter/enhancer of the steroidogenic enzymes prompted us to verify if these transcription factors are expressed in the PG. We performed *in situ* hybridization on third instar larvae and observed an intense staining of *vvl* mRNA in the PG (Fig. 1B). Moreover, strong embryonic *vvl* expression is seen in the primordium of the PG from stage 13. Importantly, the appearance of *vvl* in the PG precedes that of the biosynthetic genes which are expressed by stage 15 in the PG primordium [11], [12]. Although *in situ* expression of *kni* was undetectable in the PG of embryos, expression in the PG was observed at the L3 stage (Fig 1B). We also detected expression of *vvl* in nurse and follicle cells of adult female ovaries (Fig. S1).

Using specific antibodies, we also confirmed that Vvl and Kni are expressed in the PG and that these transcription factors localize in the nucleus (Fig. 1C). Although *kni* expression was not detected using *in situ* hybridization in the embryonic PG, expression of Kni was found in the PG at the L2 stage (Fig. S1). Next, we quantified *vvl* and *kni* expression in the ring gland (an organ with by far the most of its volume constituted by PG cells) compared to the whole body in order to see if these transcription factors are enriched in the PG (Fig. 1D). As a control, we measured *phm* expression, which indeed is highly expressed in the PG compared to the whole animal and *mld*, encoding a factor with a specific role in the PG, but with a broader expression pattern (Fig. 1D and S1) [12], [21]. Expression of *vvl* was highly enriched in the ring gland, like *phm*, while *kni* expression was less specific to this tissue similar to *mld*.

### PG loss of *vvl* and *kni* result in developmental arrest due to failure in ecdysone production

**Figure 2:**
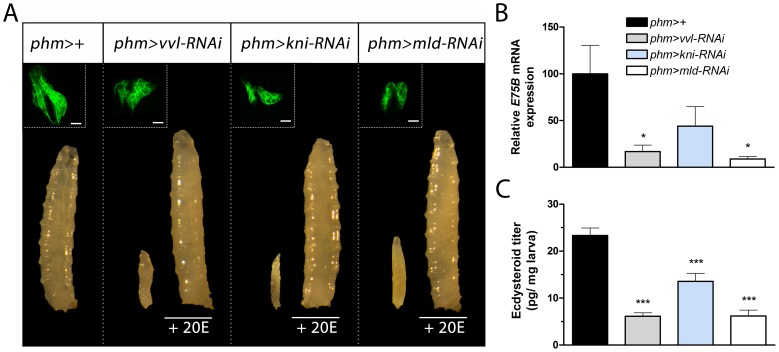
Knock down of *vvl*, *kni* and *mld* in the PG results in developmental arrest and reduces ecdysteroid levels. (A) RNAi mediated knock down of *vvl*, *kni* or *mld* in the PG using a PG specific driver (*phm*>) results in developmental L1 arrest for *phm>vvl-RNAi* and *phm>mld-RNAi* and L1 and L2 arrest for *phm>kni-RNAi* larvae. The morphology of the cells in the PG (GFP; green in the top left corner) is normal in *phm*>*GFP*,*vvl-RNAi*, *phm*>*GFP*,*kni-RNAi* and *phm*>*GFP*,*mld-RNAi* animals 36 hours AEL (scale bars, 20 µm). Supplying *phm*>*vvl-RNAi*, *phm*>*kni-RNAi* and *phm*>*mld-RNAi* larvae with 20-hydroxyecdysone (20E) rescues the developmental arrest. (B) Ecdysone levels, as measured by the ecdysone inducible gene *E75B*, is reduced in the mid-first instar (36 hours AEL) by knock down of *vvl*, *kni* or *mld* in the PG. (C) Ecdysteroid levels measured by ELISA confirm that L1 larvae with reduced expression of *vvl*, *kni* or *mld* in the PG have low levels of ecdysteroids 36 hours AEL compared to the control. Error bars indicate s.e.m. (n = 4). *P<0.05, ***P<0.001, versus the *phm*>+ control.

Based on the potential regulatory role of Vvl and Kni, we next sought to determine if these transcription factors are required for PG expression of the genes involved in ecdysone biosynthesis. We used the PG specific *phm-Gal4* (*phm>*) driver and observed that knock down of *vvl* in the PG using *UAS-vvl-RNAi* (*vvl-RNAi*) resulted in first instar (L1) arrest (Fig. 2A). Furthermore, RNAi mediated knock down of *kni* in the PG, by using *phm>* with a *UAS-kni-RNAi* (*kni-RNAi*), led to an L1 and second instar (L2) arrest phenotype. To exclude the contribution of off-target effects, we tested PG specific knock down of *vvl* and *kni* using other transgenic RNAi lines that target different regions of the *vvl* and *kni* mRNA and found that they produce similar phenotypes (Table S1). To support this, we also used the *P0206-Gal4* (*P0206>*) driver that promotes weak expression in the PG cells [22]. When expression of *vvl* and *kni* was reduced using *P0206>*, development was arrested during later stages compared to when crossed with *phm>*. Knock down of *mld* in the PG with *phm>* driven *UAS-mld-RNAi* (*mld-RNAi*) also resulted in L1 arrest (Fig. 2A) consistent with mutant analysis [9], [21].

If *kni* and *vvl* are involved in specifying the gland during embryonic development, reducing their expression may cause a lack of PG cell differentiation. We used a *phm>GFP* to label and examine the morphology of the PG in L1 larvae 36 hours after egg lay (AEL). PG cell number and morphology of L1 larvae with reduced expression of *vvl*, *kni* or *mld* in the PG were indistinguishable from the *phm>+* control (Fig. 2A). This demonstrates that knock down of these factors does not compromise PG cell fate specification and survival. The developmental arrest indicates that loss of *vvl* and *kni* in the PG impair the cellular production of ecdysone. We therefore investigated the ecdysone levels in L1 larvae 36 hours AEL by measuring *E75B* mRNA expression in the whole animal, which has been used as a readout for ecdysone levels [20], [23]. Expression of *E75B* was significantly reduced in mid-first instar *phm*>*vvl-RNAi* and *phm*>*mld-RNAi* larvae compared to the control (Fig. 2B). This is consistent with the failure of *phm*>*vvl-RNAi* and *phm*>*mld-RNAi* larvae to molt to the L2 stage. A portion of larvae with knock down of *kni* in the PG undergoes the L1–L2 transition, suggesting that some of these animals can produce sufficient ecdysone for the L1–L2 molt. Consistent with this observation, knock down of *kni* in the PG did not lead to a significant reduction of *E75B* in the mid-first instar. To demonstrate that the observed phenotypes are a result of decreased ecdysone biosynthesis, we tested if ecdysone supplementation could rescue the developmental arrest. Indeed, animals with reduced expression of *vvl*, *kni* or *mld* in the PG were rescued by the addition of 20E to their food, showing that it is the lack of this hormone that is causing the arrest (Fig. 2A). To confirm this, we measured the ecdysteroid titer, which demonstrates that larvae with reduced expression of *vvl*, *kni* or *mld* in the PG have lower levels of ecdysteroids by the mid-first instar compared with the control (Fig. 2C). Taken together, these results indicate that the transcription factors Vvl and Kni, like Mld, are required for ecdysone biosynthesis in the endocrine cells of PG during early larval development.

### Vvl and Kni are required for ecdysone production by regulating steroidogenic gene expression

**Figure 3:**
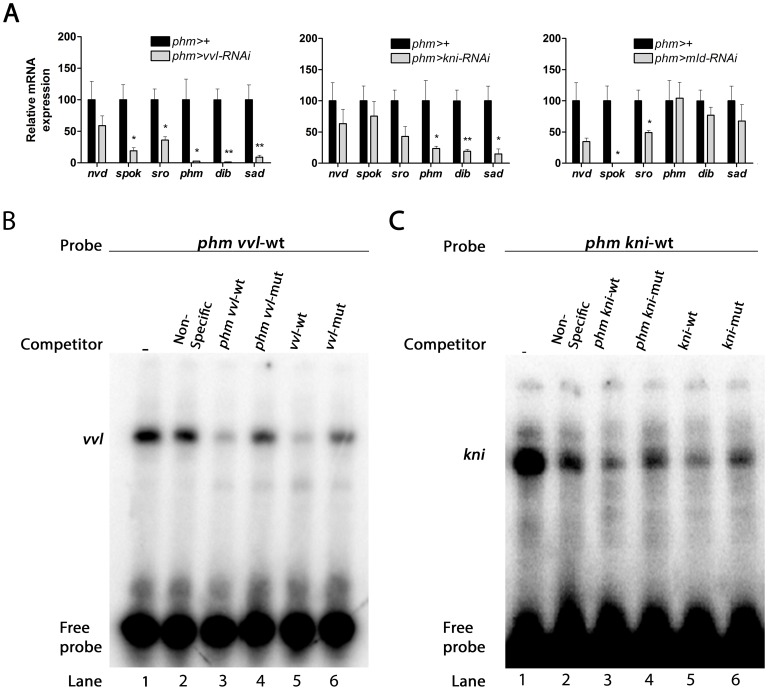
*vvl*, *kni* and *mld* are required for the expression of genes in the ecdysone biosynthetic pathway. (A) Knock down of *vvl*, *kni* and *mld* in the PG reduces expression of genes in the steroidogenic pathway. *vvl* knock down results in a down-regulation of *spok* and *sro*, catalyzing early steps in the pathway, as well as a reduction of *phm*, *dib* and *sad* mediating the last three steps in the biosynthetic pathway. Knock down of *kni* results in down-regulation of *phm*, *dib* and *sad*, while knock down of *mld* causes a specific down-regulation of *spok* and a moderate reduction of *sro*. Expression was measured in mid-first instar larvae 36 hours AEL. Error bars indicate s.e.m. (n = 4). *P<0.05, **P<0.01, versus the *phm*>+ control. (B, C) Direct binding of Vvl or Kni to the regulatory sites in *phm* promoter indicated by electrophoretic mobility shift assay (EMSA). Nuclear extract was incubated with [γ32]ATP-labeled oligonucleotide sequences of *phm* promoter containing the *vvl* (B) or the *kni* sites (C) and resulted in shifted DNA-protein bands (lane 1). Competition assays were performed with unlabeled non-specific random oligonucleotide sequences (lane 2), the *phm* promoter containing the *vvl* or *kni* sites (lane 3), the *phm* promoter with mutated *vvl* or *kni* sites (lane 4), an oligonucleotide sequence with *vvl* or *kni* consensus motif sequence (lane 5), or with the consensus motif mutated (lane 6).

We next investigated if Vvl and Kni regulate the expression of the genes involved in ecdysone biosynthesis. *phm*>*vvl-RNAi* and *phm*>*kni-RNAi* larvae showed reduction in the expression of *phm*, *dib* and *sad* by the mid-first instar 36 hours AEL compared to the control (Fig. 3A). Knock down of *vvl* also reduced expression of *sro* and *spok*, encoding enzymes believed to work in early steps in the pathway known as the black box [4], [5], [9]. However, expression of *nvd*, encoding a PG specific gene involved in the first step in the biosynthetic pathway, was not significantly reduced in the mid-first instar by knock down of *vvl* or *kni* in the PG. This further supports the notion that the PG is specified normally during embryogenesis. Previous studies have indicated that *mld* mutants have reduced ecdysone levels because of a specific lack of *spok* expression [9], [21]. Our knock down results involving *mld-RNAi* in the PG support that Mld is required specifically for the expression of *spok*, but not for the later acting products of *phm*, *dib* and *sad*.

The binding sites of these factors in the PG specific regulatory elements indicate that Vvl and Kni are involved in a transcriptional network necessary for co-expression of the biosynthetic enzymes. We therefore sought to establish if Vvl and Kni can bind directly to the PG specific regulatory elements by performing a DNA/protein binding assay. For this purpose, we performed electrophoretic mobility shift assays (EMSAs) with the conserved sites in the *phm* promoter since the functional importance of these sites has been confirmed [20]. Radiolabeled DNA oligonucleotide sequences that contained the conserved *vvl* or *kni* binding sites in the *phm* promoter required for PG expression (Fig. 1A) formed DNA/protein complexes with nuclear cell extract (Fig. 3B and C). These complexes were outcompeted by unlabeled oligonucleotide sequences containing consensus *vvl* or *kni* sites and by the unlabeled *phm* oligonucleotides containing the *vvl* or *kni* site, but not by the unlabeled *phm* oligonucleotides with mutated *vvl* or *kni* binding sites or by an unspecific oligonucleotide sequence. This finding demonstrates that the *vvl* and *kn*i sites are required for formation of the DNA/protein complex and supports that Vvl and Kni regulate transcription of the genes involved in ecdysone biosynthesis by direct binding to their promoters and enhancers.

### Vvl and kni are required to maintain expression of the biosynthetic genes during late larval development

**Figure 4:**
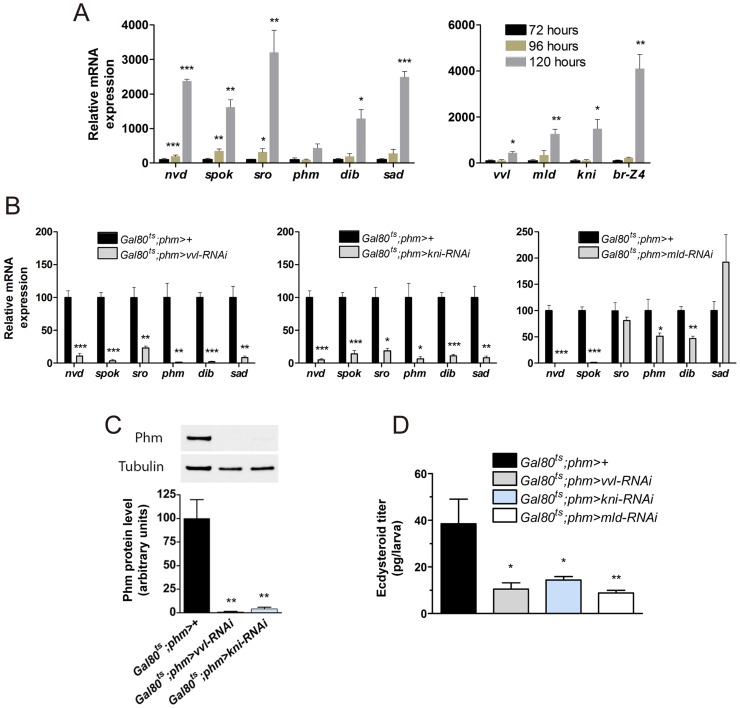
*vvl*, *kni* and *mld* have a specific role in regulating ecdysone biosynthesis in the L3 stage. (A) Expression of steroidogenic genes in ring glands from wild type (*phm-GFP*) larvae increases little from early (72 hours AEL) to mid (96 hours AEL) third instar, but rises dramatically in the late (120 hours AEL) third instar. *vvl* expression exhibits a minor increase in the late third instar, while *mld*, *kni* and especially *Br-Z4* show a strong increase (n = 4). (B) Expression in the ring gland from larvae with knock down of *vvl*, *kni* or *mld* during the L3 stage two days after temperature induced activation of the RNAi with the *Gal80^ts^*;*phm*> driver 96 hours AEL. Expression of all the steroidogenic genes were significantly reduced in animals with reduces *vvl* or *kni* expression. Knock down of *mld* results in a dramatic reduction in expression of *nvd* and *spok* that mediate two early steps in the biosynthesis of ecdysone (n = 4). (C) Quantified level of Phm protein in brain-ring gland complexes (BRGCs) from L3 larvae two days after temperature induced RNAi (96 hours AEL) normalized to Tubulin levels determined by immunoblotting (top panel) (n = 3). (D) Ecdysteroid levels determined by ELISA in L3 larvae with reduced PG expression of *vvl*, *kni* or *mld* two days after temperature induced activation of the RNAi effect (96 hours AEL) (n = 4). Error bars indicate s.e.m. *P<0.05, **P<0.01, ***P<0.001, versus the *Gal80^ts^;phm>+* control.

The data indicate that Vvl and Kni are critical for the steroidogenic activity of the PG during early post-embryonic development. Later during larval development the up-regulation of ecdysone biosynthetic genes and the growth of the gland are required to produce the high-level ecdysone pulse that triggers metamorphosis. To investigate the role of Vvl and Kni during later stages of postembryonic development, we analyzed their expression in third instar (L3) larval ring glands from early (72 hours AEL), mid (96 hours AEL) and late (120 hours AEL) L3 larvae. In wild type larvae, expression of the steroidogenic genes showed no or little increase from the early to mid L3, but a dramatic up-regulation in the late L3 (Fig. 4A), coinciding with the high-level ecdysone peak that triggers pupariation 120 hours AEL [20]. While the expression of *vvl* showed only a minor increase during the L3 stage, a stronger up-regulation of *kni* and *mld* was observed. Compared to both *vvl*, *kni* and *mld*, expression of *Br-Z4* was highly up-regulated in the late L3 consistent with its role in the temporal up-regulation of the biosynthetic genes important for the high-level ecdysone pulse 120 hours AEL that triggers pupariation [24]. Considering the tissue-specificity and that *vvl* expression in the PG shows little relation with the ecdysone titer, it seems likely that Vvl is important for the spatial control of ecdysone biosynthetic gene expression in the PG, but not for the temporal regulation during development. On the other hand, *kni* expression is less PG specific, but show more correlation with the ecdysone titer during L3.

To determine whether Vvl and Kni are only required to set the initial expression of the biosynthetic enzymes during embryonic and early larval development or also to maintain PG expression during late larval stages, we used *tub-Gal80^ts^;phm-Gal4* (*Gal80^ts^;phm>*) to conditionally induce the *UAS-RNAi* effect. *Gal80^ts^;phm>vvl-RNAi* and *Gal80^ts^;phm>kni-RNAi* larvae develop normally at 18°C and were shifted to 29°C to induce the RNAi at different times during larval stages. Development of most *Gal80^ts^;phm>vvl-RNAi, Gal80^ts^;phm>kni-RNAi* and *Gal80^ts^;phm>mld-RNAi* larvae was arrested in L3 when larvae were shifted to 29°C 120 hours AEL or earlier, while larvae that were shifted 144 hours AEL or later pupariated normally (Fig. S2). Because development is slowed down at 18°C, 120 hours AEL is corresponding to the late L2 stage under these conditions [25]. Thus, inducing RNAi in late L2 or earlier causes developmental arrest in L3, suggesting that it prevents the production of the high-level ecdysone pulse in late L3 that triggers metamorphosis.

Since expression of the biosynthetic genes detected in L1 larvae with reduced expression of *vvl* and *kni* was measured on RNA extracted from whole animals (Fig. 3A), and normalized to *Rpl23* and *Rpl32*, ubiquitously expressed ribosomal housekeeping genes, it is possible that reduced PG cell growth could be responsible for the observed decrease in biosynthetic gene expression because of a reduced PG to whole animal size ratio. To exclude this possibility and to test whether Vvl and Kni are required to maintain PG specific expression of steroidogenic genes later during development, we analyzed expression in isolated ring glands from L3 larvae. We first confirmed the efficient reduction of *vvl* and *kni* mRNA levels compared to the control (Fig. S2). Additional analysis showed that expression of all of the steroidogenic enzymes was dramatically decreased in L3 ring glands in which *vvl* and *kni* were knocked down compared to the control (Fig. 4B). This demonstrates that the decreased expression of the ecdysone biosynthetic genes in *vvl-RNAi* and *kni-RNAi* animals is a consequence of a specific reduction in the transcription of steroidogenic genes, and not reduced glandular growth or a general reduction in transcription. Compared to *vvl-RNAi* and *kni-RNAi*, *mld* knock down had little or no influence on the transcription of the genes encoding enzymes acting in late steps in the biosynthetic pathway. However, *spok* and *nvd* levels were strongly reduced in the ring glands of *mld-RNAi* larvae compared to the control, suggesting that these are direct targets of *mld* regulation. This indicates that Mld is involved in the transcriptional regulation of the enzymes mediating early biosynthetic conversions of cholesterol. The observation that *mld-RNAi* also regulates *nvd* expression may explain why *spok* overexpression in the PG is insufficient to rescue *mld* mutants [9].

To examine the influence of *vvl* and *kni* knock down on the biosynthetic enzyme level, we measured Phm protein levels in brain-ring gland complexes (BRGCs) using immunoblotting analysis. Consistent with the reduced mRNA levels, these results show that Phm protein levels are dramatically reduced in *vvl-RNAi* and *kni-RNAi* larvae compared with the control (Fig. 4C). To reinforce that knock down of *vvl, kni* or *mld* in the PG impairs ecdysone biosynthesis, we also measured the ecdysteroid levels in L3 larvae. Ecdysteroid levels were reduced in L3 larvae where RNAi mediated knock down of *vvl, kni* or *mld* in the PG had been induced in the L2 stage (Fig. 4D).

### The developmental arrest by PG inactivation of *vvl* and *kni* can be rescued by ecdysone and 20E

**Figure 5:**
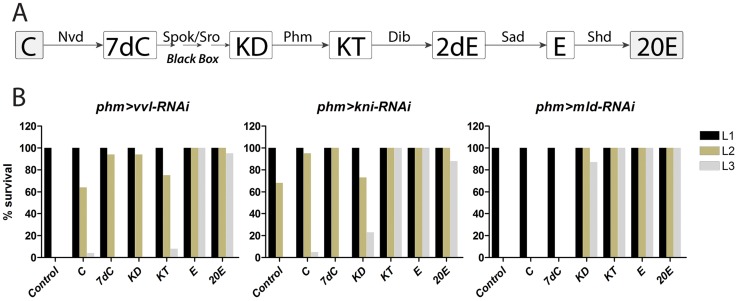
Ecdysone and 20E efficiently rescue loss of *vvl* and *kni* in the PG. (A) Ecdysone biosynthetic scheme showing steps in the conversion of cholesterol to 20-hydroxyecdysone (20E). Note that ecdysone produced and released from the PG is converted to its active form 20E in peripheral tissues. (B) Percentage of larvae developing to the indicated stage. L1; first instar larvae, L2; second instar larvae, L3; third instar larvae. Resupplying precursors later in the pathway is gradually more efficient in rescuing arrest of larvae with reduced expression of *vvl* and *kni* in the PG. In contrast, only precursors downstream of the black box efficiently rescue *mld-RNAi* larvae, indicating that Mld regulates a gene product(s) involved in the reactions upstream of the 5β-ketodiol. C; cholesterol, 7dC; 7-dehydrocholesterol, KD; 5β-ketodiol, KT; 5β-ketotriol, 2dE; 2-deoxyecdysone, E; ecdysone, 20E; 20-hydroxyecdysone.

Taken together, the data suggest that the coordinated expression of steroidogenic enzymes in the PG requires Vvl and Kni function. To further corroborate our findings that Vvl and Kni are involved in co-regulating all components in the biosynthetic pathway, we examined whether supplementation of any 20E precursors to the larval growth medium was able to rescue the developmental arrest of *vvl* or *kni* RNAi larvae. When fed cholesterol, 7-dehydrocholesterol or 5β-ketodiol, most *phm>vvl-RNAi* and *phm>kni-RNAi* animals develop to small L2 larvae (Fig. 5A and B). Since *phm>vvl-RNAi* and *phm>kni-RNAi* arrest in L1 and L2 without supplementation, it appears that increasing the amount of substrate for ecdysone synthesis provides some compensation, but not complete rescue, when the pathway activity is reduced. Supporting this notion, providing intermediates further downstream in the pathway gradually increased rescue of *phm>vvl-RNAi* and *phm>kni-RNAi* larvae to the L3 stage. In particular, 20E and its precursor ecdysone efficiently rescue *phm>vvl-RNAi* and *phm>kni-RNAi* larvae to the L3 stage (Fig. 5B).

We then tested whether increased availability of cholesterol substrate is sufficient to promote ecdysone biosynthesis. Indeed, supplementation with cholesterol increased *E75E* mRNA in wild type larvae and ecdysteroid levels in the control and in larvae with PG specific loss of *vvl*, *kni* or *mld* compared with animals grown on standard food (Fig. S3). Like rescue of the L1 arrest (Fig. 5B), cholesterol also provided minor rescue of the L3 developmental arrest observed when the RNAi effect was induced in the L2 stage (Table S2). Increasing cholesterol concentrations only provides minor rescue for loss of *vvl* and *kni*. In contrast, we confirmed that it provides complete compensation for loss of *Niemann-Pick type C-1a* (*npc1a*) (Fig. S3), which reduces substrate delivery for ecdysone biosynthesis [26], [27]. These results suggest that the hormone deficiency observed in *vvl-RNAi* and *kni-RNAi* larvae is a result of impaired ecdysone pathway activity and not compromised cholesterol substrate delivery, like in *phm>npc1a-RNAi* larvae. These findings overall indicate that silencing *vvl* or *kni* in the PG specifically inhibits synthesis of ecdysone by reducing the activity of the biosynthetic pathway.

Supplying the 5β-ketodiol and 5β-ketotriol, but not cholesterol or 7-dehydrocholesterol, rescued *mld-RNAi* larvae (Fig. 5B), consistent with Mld being required for expression of Nvd and Spok which mediate early steps in the pathway upstream of the 5β-ketodiol. We conclude, that Vvl and Kni are necessary for coordinating the tissue-specific expression of all steroidogenic genes in the endocrine cells of the PG, while Mld specifically regulates genes involved in early steps in the pathway responsible for the conversion of cholesterol to the 5β-ketodiol, an intermediate downstream of the black box reaction(s).

### PTTH and insulin/TOR signaling in the PG is disturbed by loss of *vvl* and *kni*

**Figure 6:**
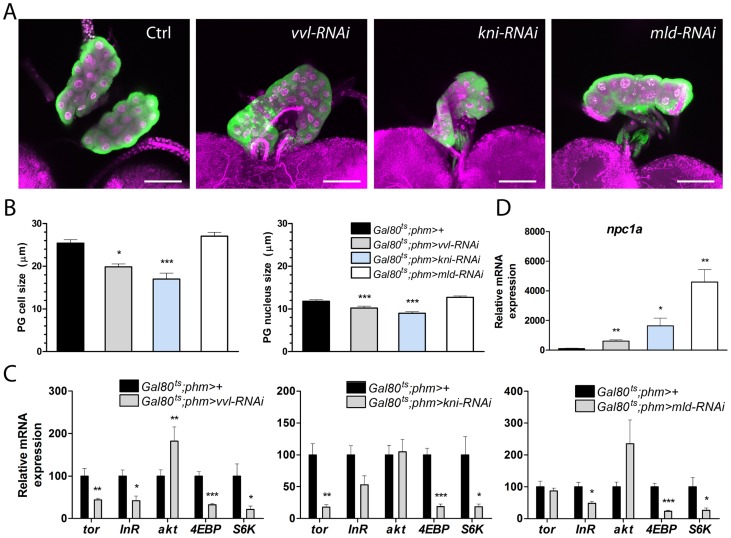
*vvl* and *kni* knock down affects PG cell size and disturbs PTTH/Torso and insulin/TOR signaling. (A, B) PG (green) and nuclei (magenta: DAPI) show that the morphology of the gland is intact in late L3 larvae with reduced expression of *vvl*, *kni*, or *mld* in the PG. PG cell and nucleus size of L3 larvae (measured as the diameter) are reduced when *vvl* and *kni* are knocked down (n = 11). RNAi was induced using the *Gal80^ts^*;*phm*>96 hours AEL corresponding to the late L2 stage at 18°C. The PG was analyzed after two days at 29°C when control larvae are in the wandering stage. (C) Knock down of *vvl* and *kni* results in decreased expression of *torso (tor)*, *4EBP* and *S6K* in dissected ring glands containing the PG. Likewise, *4EBP* and *S6K* are down regulated in ring glands of *mld-RNAi* animals that also exhibit reduced expression of the *InR*. Contrary, *akt* is increased in *vvl-RNAi* animals (n = 4). (D) Loss of *vvl*, *kni* or *mld* results in a strong increase of *npc1a* expression in the ring gland. In C and D, expression was measured in L3 larvae raised as described in A and B (n = 4). Error bars indicate s.e.m. *P<0.05, **P<0.01, ***P<0.001, versus the *Gal80^ts^*;*phm>+* control.

Our data demonstrate that Vvl and Kni are specifically involved in transcriptional regulation of ecdysone biosynthetic components. However, when we analyzed the morphology of PG cells with reduced expression of *vvl* and *kni,* we found a mild decrease in PG cell size (Fig. 6A and B), indicating that knock down of these transcription factors also influence cellular growth. The major pathways that are thought to control PG cell growth are the PTTH and the insulin/TOR pathways [22], [28]–[32]. Therefore, we investigated the possibility that Vvl and Kni affect PG cell growth and ecdysone synthesis indirectly by interfering with PTTH and/or insulin/TOR signaling. The neuropeptide, PTTH promotes PG growth and ecdysone synthesis through activation of its receptor Torso, a receptor tyrosine kinase (RTK) expressed specifically in the PG [33]. Activation of the insulin receptor (InR), another RTK, in the PG also regulates cell growth and stimulates ecdysone synthesis in response to circulating insulin levels. Although crosstalk between systemic insulin mediated growth regulation and TOR signaling might occur, the TOR pathway cell-autonomously regulates growth in response to cellular nutrient levels [34]. We therefore investigated whether PTTH and insulin/TOR signaling in the gland is affected by knock down of *vvl* and *kni*. Analysis of *torso* transcript levels revealed that, while *mld-RNAi* larvae have normal *torso* mRNA levels, expression of the PTTH receptor is reduced in ring glands from L3 *vvl-RNAi* and *kni-RNAi* larvae (Fig. 6C). Consistent with down-regulation of the PTTH receptor, we found reduced levels of phosphorylated ERK, an indicator of MAPK activity and PTTH signaling [33], in BRGCs from *vvl-RNAi* and *kni-RNAi* larvae (Fig. S4). However, unlike the biosynthetic enzymes (Fig. 3), expression of *torso* was not reduced in L1 *phm*>*vvl-RNAi* larvae 36 hours AEL (Fig. S4), indicating that *torso* expression is initiated normally despite the loss of *vvl* in the PG. When examining the expression of the *InR* and components mediating insulin signaling, we found reduced expression in *vvl-RNAi* and *kni-RNAi* animals of *4EBP* that encodes a negative growth regulator depressed by activation of the insulin pathway. Further, levels of *akt*, which encodes a serine/threonine kinase of the insulin signaling pathway [35], were increased, while levels of *InR* were decreased in *vvl-RNAi* larvae. Increased insulin signaling is generally associated with decreased expression of both *4EBP* and *InR* [36], [37]. These results imply that loss of *vvl* and *kni* increases insulin signaling. The most likely explanation for increased insulin signaling in PG of animals with reduced *vvl* and *kni* expression is the low ecdysone levels, which cause a general increase of insulin release from the brain [29]. Thus, the disturbance of insulin signaling in the PG of *vvl-RNAi* and *kni-RNAi* animals seems unlikely to account for the PG cell growth reduction. However, we observed a strong transcriptional reduction of the *S6 kinase* (*S6K*), an important positive growth regulator downstream of TOR. This suggests that the combined reduction of both PTTH/Torso and TOR/S6K signaling in the PG contributes to the negative influence of *vvl-RNAi* and *kni-RNAi* on PG cell growth and ecdysone synthesis. Why does *mld* knock down not affect PG cell size negatively (Fig. 6A and B)? Since loss of *mld* does not affect *torso* expression (Fig. 6C), it is possible that disturbance of the TOR/S6K pathway alone is insufficient to impair growth, especially if this is combined with increased insulin signaling as indicated by the decreased *InR* and *4EBP* mRNA levels in the ring glands of *mld-RNAi* larvae.

### Knock down of *vvl* and *kni* affects Npc1a involved in cholesterol trafficking

Finally, we investigated whether loss of *vvl* and *kni* in the PG affects cholesterol substrate delivery for ecdysone synthesis. Surprisingly, we found that, whereas the biosynthetic genes show a strong decrease, *npc1a* exhibits a dramatic increase in the gland of *vvl-RNAi*, *kni-RNAi* and *mld-RNAi* larvae (Fig. 6D). This finding indicates that up-regulation of *npc1a* in the PG of *vvl-RNAi*, *kni-RNAi* and *mld-RNAi* larvae reflect a compensatory feedback regulation to maintain cholesterol homeostasis and/or increase substrate delivery to promote steroidogenesis. Down-regulation of biosynthetic activity in *vvl-RNAi*, *kni-RNAi* and *mld-RNAi* larvae reduces cholesterol flux through the ecdysone pathway and may lead to intracellular redistribution of cholesterol to maintain homeostasis through feedback regulation. We therefore explored the possibility that *npc1a*, which is required for normal cholesterol distribution and availability for steroid synthesis, is controlled by feedback regulation of cholesterol. Expression of *npc1a* is repressed by cholesterol in wild type larvae (Fig. S4), indicating that *npc1a* is feedback regulated. Recently, we showed that ecdysone biosynthesis is controlled by feedback circuits in the PG [20]. We therefore also examined whether ecdysone signaling in the gland is affected by knock down *vvl*, *kni* or *mld*. To test this, we measured mRNA levels of the *ecdysone receptor* (*EcR*) ring glands isolated from L3 larvae where *vvl-RNAi*, *kni-RNAi* or *mld-RNAi* had been induced in the PG during L2. Transcript levels of *EcR* were not affected in ring glands from *vvl-RNAi* and *kni-RNAi* larvae (Fig. S4), indicating that the responsiveness of the PG to ecdysone is not reduced. Taken together, these results suggest alterations of cholesterol uptake and trafficking in the PG when flow through the biosynthetic pathway is impaired.

## Discussion

*Drosophila* developmental progression is dictated by tightly regulated ecdysone pulses released from the PG. Like any cell specialized for steroid biosynthesis, the PG expresses a set of enzymes that mediate steps in the conversion of cholesterol into steroids. The tissue-specific expression of these enzymes is key to the specialization of the cells that endows the PG with the competence to produce ecdysone. The transcriptional control mechanism underlying such regulation is likely orchestrated by a regulatory network of transcription factors. Here, we identify two transcription factors Vvl and Kni that are required for the expression of the biosynthetic enzymes in the ecdysone producing PG cells. Vvl is a POU domain transcription factor which has multiple important functions during *Drosophila* development. Mutations in *vvl* cause embryonic lethality with defects in the development of the trachea and the nervous system [38]–[41]. Moreover, Vvl is required for wing vein development and is involved in innate immunity by regulation of the expression of antimicrobial peptides [42], [43]. We show that Vvl is expressed in the PG during late embryogenesis and in the larval stages. One important characteristic of Vvl is that it maintains its own expression by autoregulation [44]. Once activated, Vvl maintains its expression and likely also the expression of the ecdysone biosynthetic genes in the PG. Knock down of *vvl* in the PG reduces the expression of all genes in the biosynthetic pathway, showing that Vvl is required for maintaining expression of all pathway components. Together with the high expression of Vvl in the gland, this suggests that Vvl is a master transcriptional regulator involved in specifying the genetic program that dictates PG cell identity including its tissue-specific expression of steroidogenic enzymes. It is interesting to note that human chromosome 6 deletions that affect POU3F2, a homolog of Vvl, have been associated with hypogonadotropic hypogonadism and adrenal insufficiency [45], [46], making it possible that Vvl is a conserved regulator of steroid biosynthesis.

The gap gene *kni* is known for its role in embryonic segmentation patterning and development of the trachea and wing vein [47]–[51] similar to *vvl*. Kni is a nuclear receptor with a zinc-finger motif that is unlikely to be ligand activated since it lacks a ligand-binding domain. Our data show that Kni is required for expression of the genes involved in ecdysone biosynthesis in the PG, suggesting that Kni functions as an activator in this situation. Although Kni is generally considered a short-range repressor [52], it is required to activate hairy expression in stripe 6 during embryogenesis [53]. Thus, Kni may act either as a repressor or as an activator in a context-dependent manner. In mammals, nuclear receptors are also key regulators of steroidogenic target genes encoding P450 enzymes [54]–[56].

Although Vvl and Kni specifically control genes in the steroidogenic pathway, other targets of these factors could also be important for ecdysone synthesis in the PG. During development the continuous growth of the PG cells and endoreplication of DNA is important to scale its hormone production to the capacity required for developmental progression. We found that both *vvl-RNAi* and *kni-RNAi* larvae have mildly reduced PG nuclei and cell size, which is likely to contribute to the reduced ecdysone levels in these animals. Kni has been shown to suppress endoreplication activity in the gut by regulating cell cycle genes [48]. This is in contrast to our observation indicating that loss of *kni* results in a reduction in the nuclei size, and hence, reduced polyploidy of the PG cells. Instead our results indicate that loss of *vvl* and *kni* reduces activity of PTTH/Torso and TOR/S6K signaling, two major pathways that promote growth and stimulate ecdysone biosynthesis [30], [31], [33], [57]. However, loss of *vvl* and *kni* had no effect on *torso* expression in the mid-first instar. This indicates that these factors are not required for the initial setting of *torso* expression, but for the maintenance of high *torso* expression during development. In tracheal cells, Vvl is required to maintain expression of the RTK *breathless*, but not for activating its initial expression [42], [58]. It is unclear how the transcription of the biosynthetic enzymes fluctuates during the low level ecdysone peaks in L1 and L2, before the induction of the steroidogenic pathway by PTTH stimulation [17]. Unlike PTTH/Torso, Vvl and Kni are required in the PG during L1 and L2 for the transition to the L3 stage, which suggests that Vvl and Kni are important for the proper transcription of the biosynthetic enzymes throughout larval development. Altogether, these data suggest that in addition to being required to initiate and maintain expression of the biosynthetic enzymes, Vvl and Kni play an indirect role important for ecdysone production by enabling PG cells to be competent to respond to PTTH and by regulating the TOR/S6K pathway. In contrast, Vvl and Kni are not required for normal expression of *EcR* in the gland, indicating that feedback regulation of ecdysone biosynthesis is not influenced by knock down of these factors [20]. In contrast to the transcription factor *Br-Z4* involved in positive feedback regulation, which is strongly induced in the PG during late L3 to up-regulate expression of the biosynthetic pathway components, PG expression of *vvl* shows little relation with the high-level ecdysone peak that triggers pupariation. Taken together these data suggest that Vvl is required for maintaining PG specific expression (i.e. spatial control), while temporal regulation during development is controlled by other factors such as Br-Z4. Furthermore, our results confirm that Mld is required for PG expression of *spok* [9], but we also found that it controls Nvd, an enzyme that acts upstream of Spok in the biosynthetic pathway [4]. Thus, our data suggest that Mld is a specific regulator of the two early enzymes Nvd and Spok, while its function is not important for biosynthetic reactions that are downstream of Spok and the black box reaction(s) and the responsiveness of the PG to PTTH.

Our data show that Vvl and Kni are required in the PG during post-embryonic development to maintain PG specific expression of the ecdysone biosynthetic genes. During embryogenesis, *vvl* expression appears in the PG primordium by stage 13, after the embryonic ecdysone pulse (stage 8–12 [12]) that is required for morphogenesis and differentiation of the embryo. During early embryonic development where the PG primordium is not yet formed, the spatial expression patterns of *kni* and *vvl* (Fig. 1B) are different from the biosynthetic genes essential for the embryonic ecdysone pulse [8], [9], [11], [12], [59]. This suggests that Vvl and Kni regulate the biosynthetic genes in the PG, but not during early embryonic development. Consistent with this notion, *vvl* and *kni* mutants differentiate the embryonic cuticle [42], [60], unlike the ecdysone deficient mutants that are unable to produce the embryonic ecdysone peak [11], [59]. In adult females, the ovaries are believed to be the source of ecdysone consistent with expression of the ecdysone biosynthetic genes in the nurse and/or follicle cells [11]–[13]. In adult females, we find that *vvl* is expressed in both nurse and follicle cells, suggesting that Vvl may be involved in regulating expression of the ecdysone biosynthetic genes in the adult stage.

Interestingly, we observed that loss of *vvl*, *kni* or *mld* results in dramatic increase of *npc1a* expression in the PG. Npc1a is highly expressed in the PG where it is required for uptake and intracellular trafficking of cholesterol for steroidogeneis [26]. Larvae with loss of *npc1a* exhibit a punctuate pattern of sterol accumulation in the PG cells, indicating defects in cholesterol transport within the cells. Normally cholesterol is taken up as low density lipoproteins (LDLs) and trafficked within endosomes to the lysosomes where hydrolysis releases free cholesterol that is delivered to the plasma membrane and endoplasmic reticulum (ER) [61] where the first step in the conversion of cholesterol to ecdysone likely takes place. Why is *npc1a* up-regulated in the PG when ecdysone synthesis and pathway activity is impaired? It seems unlikely that Vvl, Kni and Mld are all involved in repression of *npc1a*. The block of flux through the biosynthetic pathway in the PG of *vvl-RNAi*, *kni-RNAi* and *mld-RNAi* animals may change intracellular cholesterol pools in the gland and affect feedback regulation to maintain cholesterol homeostasis. Our results indicate that *npc1a* is regulated by cholesterol suggesting that the up-regulation of *npc1a* may be part of a feedback regulatory response to changes in cellular cholesterol levels. This may indicate a compensatory mechanism to redistribute cholesterol by increasing storage of cholesterol esters and/or efflux to reduce free cholesterol levels when ecdysone biosynthesis is blocked. Moreover, *npc1a* is regulated by Br [27], a factor induced by EcR in the PG [20], implying that *ncp1a* may also be regulated by ecdysone feedback. Our study shows that cholesterol availability is an important parameter for ecdysone biosynthesis. Interactions between cholesterol and ecdysone feedback mechanisms may therefore be important for coordinating the supply cholesterol with the rate of steroidogenesis.

A key aspect of steroidogenesis is regulating the tissue-specific expression of the biosynthetic enzymes. We have shown here that the transcription factors, Vvl and Kni, are required for the coordinated expression of ecdysone biosynthetic genes in the PG. The transcriptional activation by Vvl and Kni is likely mediated by direct binding to cis-regulatory elements responsible for PG specific expression. This identifies an important new role for Vvl and Kni during post-embryonic development in the gene regulatory network of the steroid hormone producing cells in *Drosophila*.

## Materials and Methods

### *Drosophila* strains and husbandry

The following *Drosophila* strains were used in this study: *w^1118^, UAS-vvl-RNAi* (#110723), *UAS-kni-RNAi* (#2980), *UAS-mld-RNAi* (#101867) and *UAS-npc1a-RNAi* (#105405) from the Vienna *Drosophila* RNAi Center (VDRC); *UAS-vvl-RNAi* (#26228), *UAS-kni-RNAi* (#34705), *tub-Gal80^ts^* and *UAS-CD8-GFP* (*UAS-GFP*) from the Bloomington *Drosophila* Stock Center (BDSC); *phm^22^-Gal4* (*phm-Gal4*) [9] and *P0206-Gal4* [29]. A transgenic line *phm-291-4B* (*phm-GFP*) with a 69 bp *phm* promoter in a pH-stinger GFP reporter vector generated in [20] was used to collect ring glands by dissection for analyzing the development expression profile in the gland. Flies were raised on standard cornmeal food under a 12∶12 hour light:dark cycle. For experiments involving staged or timed larvae, flies were allowed to lay eggs at 25°C for 2–4 hours on apple juice agar plates supplemented with yeast paste in a humidified chamber. After 24 hours, 25 L1 larvae were collected and transferred to vials containing standard food. For experiments using *tub-Gal80^ts^*, eggs deposited at 25°C were immediately transferred to 18°C and 25 larvae were transferred to vials containing food 48 hours later. Images of phenotypes were captured with an Olympus SZX7 camera and analyzed using AxioVision software (Zeiss). Characterization of the PG-specific *spok* element was done as described [20] by generating transgenic animals with constructions of 5′-UTR spok elements in a pH-stinger GFP reporter vector.

### Electrophoretic mobility shift assay (EMSA)

EMSA was carried out as previously described [20]. DNA oligonucleotide sequences (Table S3) were designed to cover Vvl and Kni binding sites in the *phm* promoter based on *in silico* analysis using Transfac and Jaspar databases. Oligos containing Vvl (Vvl-wt) or Kni (Kni-wt) consensus binding sites and oligos with mutations that disrupt the Vvl (Vvl-mut) or Kni (Kni-mut) binding sites were adapted from [62], [63]. The complementary oligonucleotides were annealed and labeled at the 5′-end labeling by [γ^32^P]ATP (Perkin Elmer) using T4 polynucleotide kinase (Fermentas) and purified using Microspin G-25 columns (GE Healthcare). The EMSA reaction was performed on ice by mixing *Drosophila* S2 cell nuclear extracts (Active Motif), dialysis buffer (25 mM Hepes pH 7.6, 40 nM KCl, 0.1 mM EDTA, 10% glycerol), gelshift buffer (25 mM Tris-HCl pH 7.5, 5 mM MgCl_2_, 60 mM KCl, 0.5 mM EDTA, 5% Ficoll 400, 2.5% glycerol, 1 mM DTT and protease inhibitors) and poly(dI-dC) (Invitrogen). The reaction mixture was supplemented and incubated with 25-50-fold molar excess of unlabeled competitor nucleotides before adding the radiolabeled probe. After incubation the mixture was supplemented with gelshift loading buffer and run on a 5% non-denaturing polyacrylamide gel and dried on a slab gel dryer (Savant) followed by exposure onto a phosporimager screen. The image was acquired using a Storm 840 scanner (Molecular Dynamics) and processed with ImageQuant software version 5.2.

### Immunostaining

Tissue dissections were performed in PBS followed by fixation in 4% formaldehyde for 20 minutes at room temperature. For this study, the following primary antibodies were: mouse anti-GFP 1∶200 (Clontech, #632380); rabbit anti-Phm 1∶200 [18]; rat anti-Kni, 1∶1000 [64] and rat anti-Vvl 1∶1000 [65]. Tissues were incubated over night with primary antibodies at 4°C. Fluorescent conjugated secondary antibodies used were goat anti-mouse Alexa Fluor 488 (A11001, Invitrogen), goat anti-rabbit Alexa Fluor 555 (A21429, Invitrogen) and goat anti-rat Alexa Fluor 555 (A21434, Invitrogen). Secondary antibodies were diluted 1∶200 and incubated for two hours at room temperature. DAPI was used in 1∶500 for nuclei staining. Confocal images were captured using Zeiss LSM 710 laser scanning microscope and processed using ImageJ (NIH). Images of mid-first instar PG morphology were obtained by confocal imaging of live L1 larvae (36 hours AEL) mounted in 80% glycerol.

### Feeding experiment with steroids and precursors

Preparation and synthesis of 3β,14α-Dihydroxy-5β-cholest-7-en-6-one (5β-ketodiol) and 3β,14α,25-Trihydroxy-5β-cholest-7-en-6-one (5β-ketotriol) were previously described [8]. For the steroid feeding rescue experiment, 30 mg of dry yeast was mixed with 57 µl H_2_O and 3 µl ethanol or supplemented with 3 µl of the following sterols dissolved in ethanol: 20E (Sigma; 450 µg), ecdysone (Sigma; 100 µg), cholesterol (Sigma; 45 µg), 7-dehydrocholesterol (Sigma; 200 µg), 5β-ketodiol (450 µg), or 5β-ketotriol (280 µg). Thirty larvae were transferred to the yeast paste on an apple juice agar plate and allowed to develop in a humid chamber at 25°C. The phenotype of the larvae was scored at day 5 prior to pupariation of *w^1118^* control for rescue to the L3 stage. For other experiments with cholesterol supplementation of the food, standard cornmeal was supplied with cholesterol (Sigma) dissolved in ethanol to a final concentration of 40 µg/ml.

### *In situ* hybridization

Digoxigenin (DIG)-labeled antisense RNA probes were synthesized using DIG RNA labeling mix (Roche) and T3 (Fermentas), T7 (Fermentas) or SP6 (Roche) RNA polymerase according to the manufacturer's instructions. For the *kni* probe, an EST clone GH19318 [66] was used as a templates. For the *vvl* probe, a portion of *vvl* gene was amplified by PCR with cDNA derived from *w^1118^* larvae and the following primers: vvl_PA_CDS_F (5′-ATGGCCGCGACCTCGTACATGAC-3′) and vvl_PA_CDS_R (5′-CTAGTGGGCCGCCAACTGATGC-3′). For the *mld* probe, a portion of *mld* gene was amplified by PCR with the plasmid *mld*-pUAST [21]; a gift from S. M. Cohen and the following primers: mld_CDS_1_F (5′-ATGAGTGCCAACCGAAGAAGACG-3′) and mld_CDS_1_R (5′-CATCTGAGATTGGTCATGAGATTGTACTTGAGG-3′). PCR products containing the *vvl* and *mld* fragments were subcloned into *Sma*I-digested pBluescript II SK(-) and pCRII-Blunt-TOPO (Invitrogen), respectively, and then used as the templates for synthesizing RNA probes. Fixation, hybridization and detection were performed as previously described [8], [67].

### Quantitative RT-PCR

For gene expression experiments using the whole animals, 30 L1 larvae or 4 L3 larvae were used for each replicate. For analysis of ring gland expression, 10–15 ring glands were dissected in PBS and directly transferred to RNA lysis buffer. RNA was extracted using the RNeasy mini kit (Qiagen) and DNase treated to avoid genomic DNA contamination according to the manufacturer's instructions. RNA was quantified using a NanoDrop (Thermo Scientific) and the integrity was assessed using agarose gel electrophoresis. Total RNA was used for cDNA synthesis with the SuperScript III First-Strand Synthesis kit (Invitrogen). Primers were designed using the Primer3 software [68] (Table S4). Relative gene expression was analyzed using a Mx3000P qPCR System (Agilent Technologies) with the QuantiTect SYBR Green PCR Kit (Qiagen) according to the manufacturer's instructions as described [10], [33], [69]. All reactions were subjected to 95°C for 10 min, followed by 45 cycles of 95°C for 15 sec, 60°C for 15 sec and 72°C for 15 sec. Dissociation curve analysis was applied to all reactions to ensure the presence of single specific PCR product. Non-reverse transcribed template controls and non-template controls were included to check for background and potential genomic contamination. No product was observed in these reactions. Efficiencies were calculated for each primer pair from standard curves generated from serial dilutions of a mix of cDNA from all control samples. PCR efficiencies were always close to 100%, which was therefore used as the standard in all calculations. Expression of target genes was normalized to reference gene, *Rpl23* and *Rpl32*. We confirmed that these reference [32], [33], [70]–[74] are stably expressed across tissues and experimental conditions, by comparing *Rpl23* and *Rpl32* mRNA levels in cDNA synthesized from equal amounts of RNA extracted from different tissues and developmental stages (Fig. S2). Reference gene stability determined using qBASE Plus (Biogazelle NV, Zwijnaarde, Belgium) was within the recommended limits (M = 0.274 and CV = 0.095). For definition of these stability factors see [75].

### Ecdysteroid measurements

For ecdysteroid measurements, ecdysteroids were extracted from whole animals as described [24]. Briefly, whole larvae were rinsed in water and stored at −80°C. Samples were homogenized in 0.5 ml methanol and the supernatant was collected following centrifugation at 14,000 g. The remaining tissue was re-extracted first in 0.5 ml methanol then in 0.5 ml ethanol. The pooled supernatants were evaporated using a SpeedVac and redissovled in ELISA buffer (1 M phosphate solution, 1% BSA, 4 M sodium chloride and 10 mM EDTA). ELISA was performed according to the manufacturer's instructions using a commercial ELISA kit (ACE Enzyme Immunoassay, Cayman Chemical) that detects ecdysone and 20-hydroxyecdysone with the same affinity [76]. Standard curves were generated using 20E (Sigma). Absorbance was measured at 405 nm on a plate reader, ELx80 (BioTek) using the Gen5 software (BioTek).

### Western blotting

Four brain-ring gland complexes were dissected in cold PBS and transferred to 20 µl Laemmli Sample Buffer (Bio-Rad) supplemented with 2-mercaptoethanol. Samples were boiled for 5 minutes, centrifuged at 14,000 g and 10 µl supernatant were loaded on a 4–20% polyacrylamide gradient gel (Bio-Rad) followed by transfer onto a PVDF membrane (Millipore). Primary antibodies used were; mouse anti-α-tubulin, 1∶5,000 (T9026, Sigma Aldrich), rabbit anti-Phm, 1∶1,000 [18] and rabbit anti-phospho-ERK, 1∶1,000 (9101, Cell Signaling Technology). Secondary antibodies were goat anti-mouse IRDye 680RD, 1∶10,000 (926-68070, LI-COR) and goat anti-rabbit IRDye 800CW, 1∶10,000 (926–32211, LI-COR). The blot was scanned on an Odessey Fc (LI-COR) and the software, Image Studio for Odessey Fc, was used for image processing and protein quantification.

### Statistics

The statistical differences between data sets were calculated using two-tailed Student's t-test and error bars represent standard error of the mean (s.e.m.).

## Supporting Information

Figure S1 PG-specific cis-regulatory elements of *spok*, *phm* and *dib*, immunostaining and *in situ* hybridization. (A) Vvl and Kni binding sites are indicated on the promoter and enhancer sequences. (B) Immunostaining of the PG from an L2 larva with antibodies against Kni (magenta) and Phm (green). Scale bars, 25 µm. (C) Staining with an antisense *mld* probe indicates expression of *mld* in the ring gland PG cells of L3 larvae (*b′*), but no staining was observed in the embryonic PG (*a′*). (D) *In situ* hybridization of adult female ovaries with antisense probes for *vvl* indicate strong staining in the nurse cells and weaker staining in the follicle cells.
(TIF)

Figure S2 Effect of inducing the RNAi at different times during development, RNAi knock down efficiency and reference gene stability. RNAi mediated knock down of *vvl, kni* or *mld* was induced at different times using (A) *tubGal80^ts^;phm-Gal4* (*Gal80^ts^;phm*>) or (B) *tubGal80^ts^;phm-Gal4,UAS-GFP* (*Gal80^ts^;phm*>*GFP*) by shifting larvae from 18°C to 29°C at the indicated times. (A) Inducing the RNAi effect until 120 hours AEL at 18°C blocks pupariation, while shifting larvae 144 hours and later has little influence on pupariation. This indicates that inducing knock down by *Gal80^ts^;phm*> of *vvl*, *kni* and *mld* as late as 120 hours AEL reduces ecdysone biosynthesis and prevents formation of the high level pulse that triggers pupariation. (B) The effect is strongest when inducing the RNAi 96 hours AEL with the *Gal80^ts^;phm>GFP* driver including GFP. To facilitate analysis of the ring gland, we chose to use *Gal80^ts^;phm>GFP* that labels the PG by expression of GFP (for simplicity hereafter referred to as *Gal80^ts^;phm>*) for all further experiments. (C) Knock down efficiency of *vvl*, *kni* and *mld* in the PG. When the RNAi was induced in the PG 96 hours AEL, expression of *vvl* and *kni* was reduced to 20 or 10 percent, respectively, in dissected ring glands two days later, at the time when the control larvae were in the wandering stage. Expression of *mld* was reduced to 50 percent at this time. Black bars are the control (*Gal80^ts^*;*phm*>) and gray bars show the indicated RNAi animals (n = 5). *P<0.05, **P<0.01, versus the *Gal80^ts^*;*phm*>+ control. (D) Stability of reference gene expression in different stages and tissues. Expression of the reference genes *Rpl23* and *Rpl32* in first instar (L1) and third instar (L3) whole larvae shows that these reference genes are stably expressed in the different developmental stages analyzed. Comparison of *Rpl23* and *Rpl32* relative quantities in the ring gland (prothoracic gland) of L3 larvae and whole L3 larvae shows stable expression of these genes. Error bars indicate s.e.m.
(TIF)

Figure S3 Effect of a high-cholesterol diet on ecdysteroid levels. (A) Effect of substrate concentrations on ecdysone biosynthesis was determined by measuring the transcription of *E75B*, as a proxy for ecdysone levels. Expression of *E75B* was determined in wild type (*w^1118^*) late L3 larvae 120 hours AEL, grown either on standard food (−) or a high-cholesterol diet (+). Elevated *E75B* expression in L3 larvae grown in the presence of cholesterol indicates that the amount of ecdysone produced depends on the supply of substrates (n = 4). **P<0.01, versus the control grown on standard food. (B) RNAi was induced in larvae 96 hours AEL by switching larvae from 18°C to 29°C and ecdysteroid levels were analyzed 36 hours later when control (*Gal80^ts^;phm*>+) larvae raised on high cholesterol exhibited wandering behavior, while animals raised on a standard diet were still in the pre-wandering stage. Ecdysteroid levels are increased in larvae raised on a high-cholesterol diet compared to standard food conditions, consistent with the accelerated development, indicated by the wandering behavior normally associated with the high-level ecdysone peak [24] (n = 4). *P<0.05, ***P<0.001, versus the *Gal80^ts^;phm>+* control. (C) The PG must take up cholesterol from circulation to support ecdysone synthesis, a process that requires the function of Npc1a [26]. *phm>npc1a-RNAi* animals, with impaired delivery of cholesterol for ecdysone biosynthesis, arrest development in L1 when grown on a standard diet, but develop normally when cholesterol is increased by dietary supplementation. Error bars indicate s.e.m.
(TIF)

Figure S4 Effect of loss of *vvl* and *kni* on PTTH and ecdysone signaling and cholesterol feedback regulation of *npc1a*. (A) Quantification of phosphorylated ERK (p-ERK) levels in brain-ring gland complexes (BRGCs) from L3 animals determined by immunoblotting with an antibody specific for p-ERK. RNAi was induced 96 hours AEL by switching larvae from 18°C to 29°C and p-ERK levels were measured in BRGCs two days later. p-ERK levels were normalized to Tubulin. *P<0.01, versus the *Gal80^ts^*;*phm*>+ control. (B) Expression of *torso* was analyzed 36 hours AEL in mid-first instar larvae. (C) mRNA levels of *npc1a* were measured in wild type (*w^1118^*) L3 larvae 120 hours AEL, grown either on standard food (−) or on a high-cholesterol diet (+). Expression of *npc1a* was repressed by cholesterol (n = 4). **P<0.01, versus the control grown on standard food. (D) Expression of *EcR* in ring glands from L3 larvae two days after temperature induced activation of *vvl-RNAi*, *kni-RNAi* or *mld-RNAi* in the PG by switching larvae 96 hours AEL from 18°C to 29°C (n = 5). *P<0.01, versus the *Gal80^ts^*;*phm*>+ control. Error bars indicate s.e.m.
(TIF)

Table S1 Phenotypes with different RNAi lines using the strong *phm>* and weaker *P0206>* PG drivers. Crosses using the weak *P0206>* were raised at 29°C to enhance the activity of the Gal4/UAS system. Note that the *vvl-RNAi #110723* and the *kni-RNAi #34705* lines were used for all experiments unless otherwise stated. VDRC (Vienna *Drosophila* RNAi Center), BDSC (Bloomington *Drosophila* Stock Center).
(DOCX)

Table S2 Development of *vvl-RNAi*, *kni-RNAi* and *mld-RNAi* larvae grown on a normal diet or on a high cholesterol diet. RNAi was induced 96 hours AEL by switching L2 larvae from 18°C to 29°C.
(DOCX)

Table S3 List of oligos used for EMSA. Mutations introduced are underlined.
(DOCX)

Table S4 List of primers used for qPCR. *Primers were adapted from [32].
(DOCX)
