# Supplementary figures and images for: Transcriptional Control of Steroid Biosynthesis Genes in the Drosophila Prothoracic Gland by Ventral Veins Lacking and Knirps
Source: PLoS Genet. 2014 Jun 19;10(6):e1004343. doi: 10.1371/journal.pgen.1004343 (PMC4063667; doi:10.1371/journal.pgen.1004343)

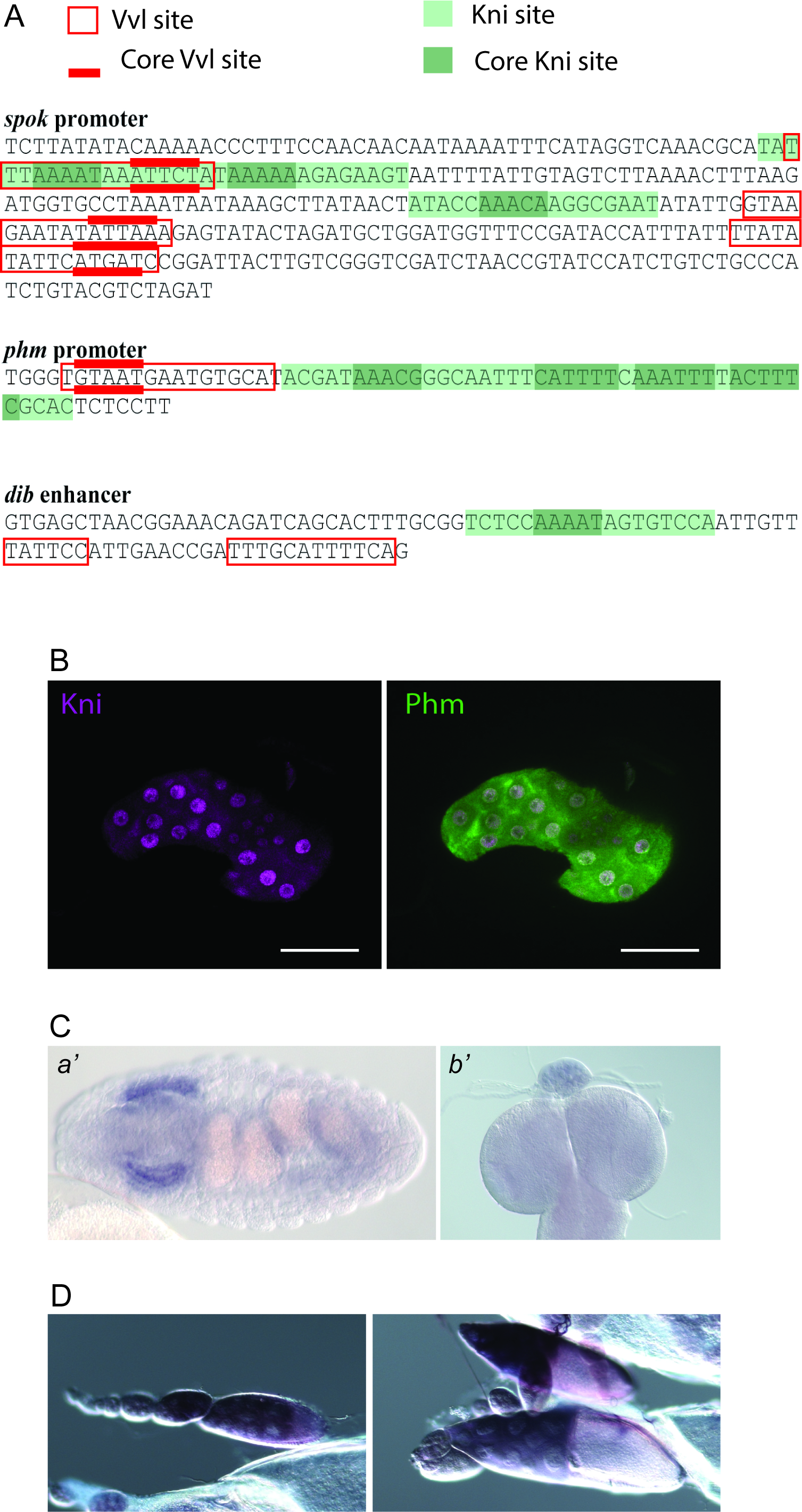

Supplement: Figure S1 — PG-specific cis-regulatory elements of spok, phm and dib, immunostaining and in situ hybridization. (A) Vvl and Kni binding sites are indicated on the promoter and enhancer sequences. (B) Immunostaining of the PG from an L2 larva with antibodies against Kni (magenta) and Phm (green). Scale bars, 25 µm. (C) Staining with an antisense mld probe indicates expression of mld in the ring gland PG cells of L3 larvae (b′), but no staining was observed in the embryonic PG (a′). (D) In situ hybridization of adult female ovaries with antisense probes for vvl indicate strong staining in the nurse cells and weaker staining in the follicle cells. (TIF) [file pgen.1004343.s001.tif]

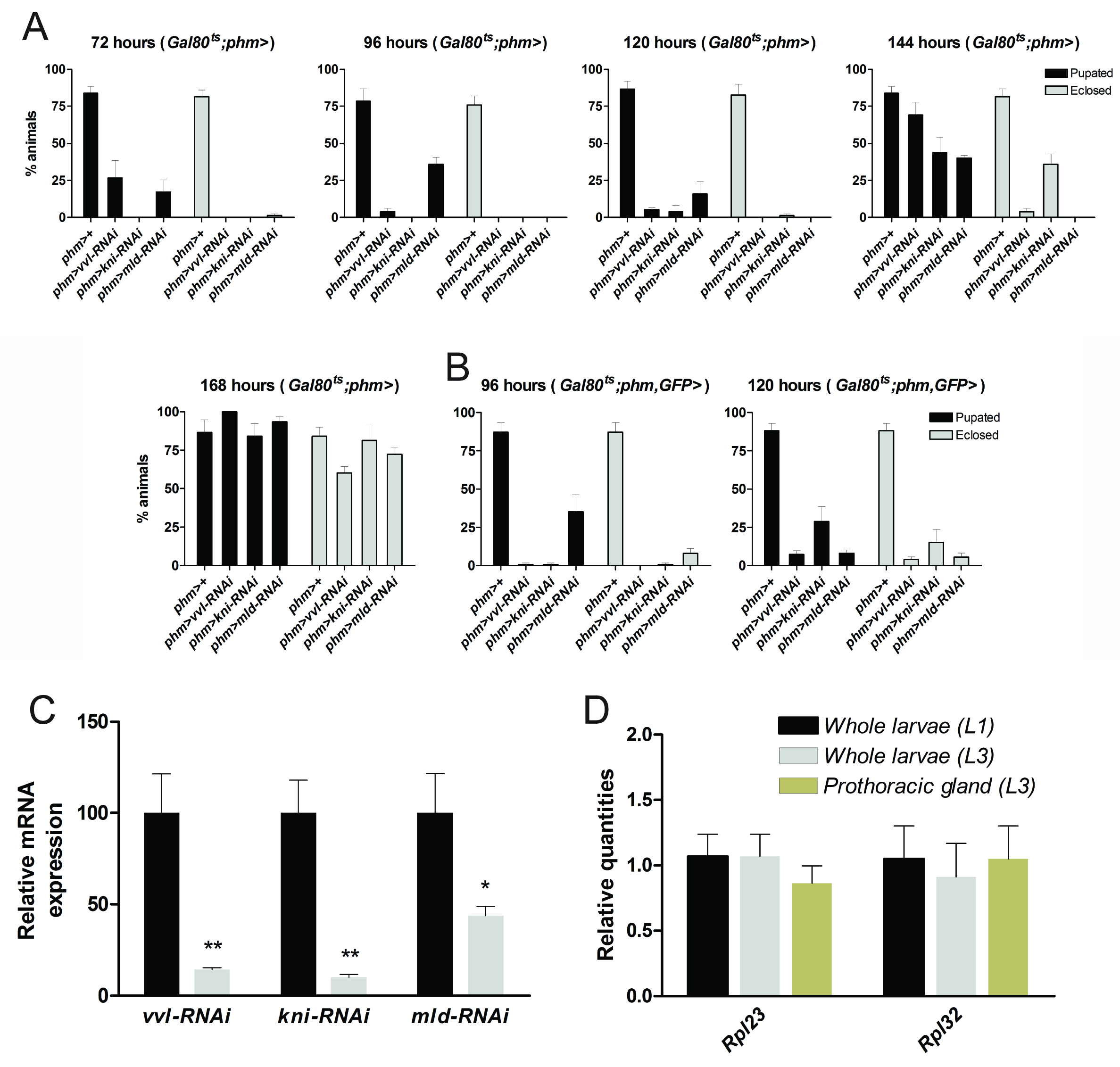

Supplement: Figure S2 — Effect of inducing the RNAi at different times during development, RNAi knock down efficiency and reference gene stability. RNAi mediated knock down of vvl, kni or mld was induced at different times using (A) tubGal80ts;phm-Gal4 (Gal80ts;phm>) or (B) tubGal80ts;phm-Gal4,UAS-GFP (Gal80ts;phm>GFP) by shifting larvae from 18°C to 29°C at the indicated times. (A) Inducing the RNAi effect until 120 hours AEL at 18°C blocks pupariation, while shifting larvae 144 hours and later has little influence on pupariation. This indicates that inducing knock down by Gal80ts;phm> of vvl, kni and mld as late as 120 hours AEL reduces ecdysone biosynthesis and prevents formation of the high level pulse that triggers pupariation. (B) The effect is strongest when inducing the RNAi 96 hours AEL with the Gal80ts;phm>GFP driver including GFP. To facilitate analysis of the ring gland, we chose to use Gal80ts;phm>GFP that labels the PG by expression of GFP (for simplicity hereafter referred to as Gal80ts;phm>) for all further experiments. (C) Knock down efficiency of vvl, kni and mld in the PG. When the RNAi was induced in the PG 96 hours AEL, expression of vvl and kni was reduced to 20 or 10 percent, respectively, in dissected ring glands two days later, at the time when the control larvae were in the wandering stage. Expression of mld was reduced to 50 percent at this time. Black bars are the control (Gal80ts;phm>) and gray bars show the indicated RNAi animals (n = 5). *P<0.05, **P<0.01, versus the Gal80ts;phm>+ control. (D) Stability of reference gene expression in different stages and tissues. Expression of the reference genes Rpl23 and Rpl32 in first instar (L1) and third instar (L3) whole larvae shows that these reference genes are stably expressed in the different developmental stages analyzed. Comparison of Rpl23 and Rpl32 relative quantities in the ring gland (prothoracic gland) of L3 larvae and whole L3 larvae shows stable expression of these genes. Error bars indicate s.e.m. (TIF) [file pgen.1004343.s002.tif]

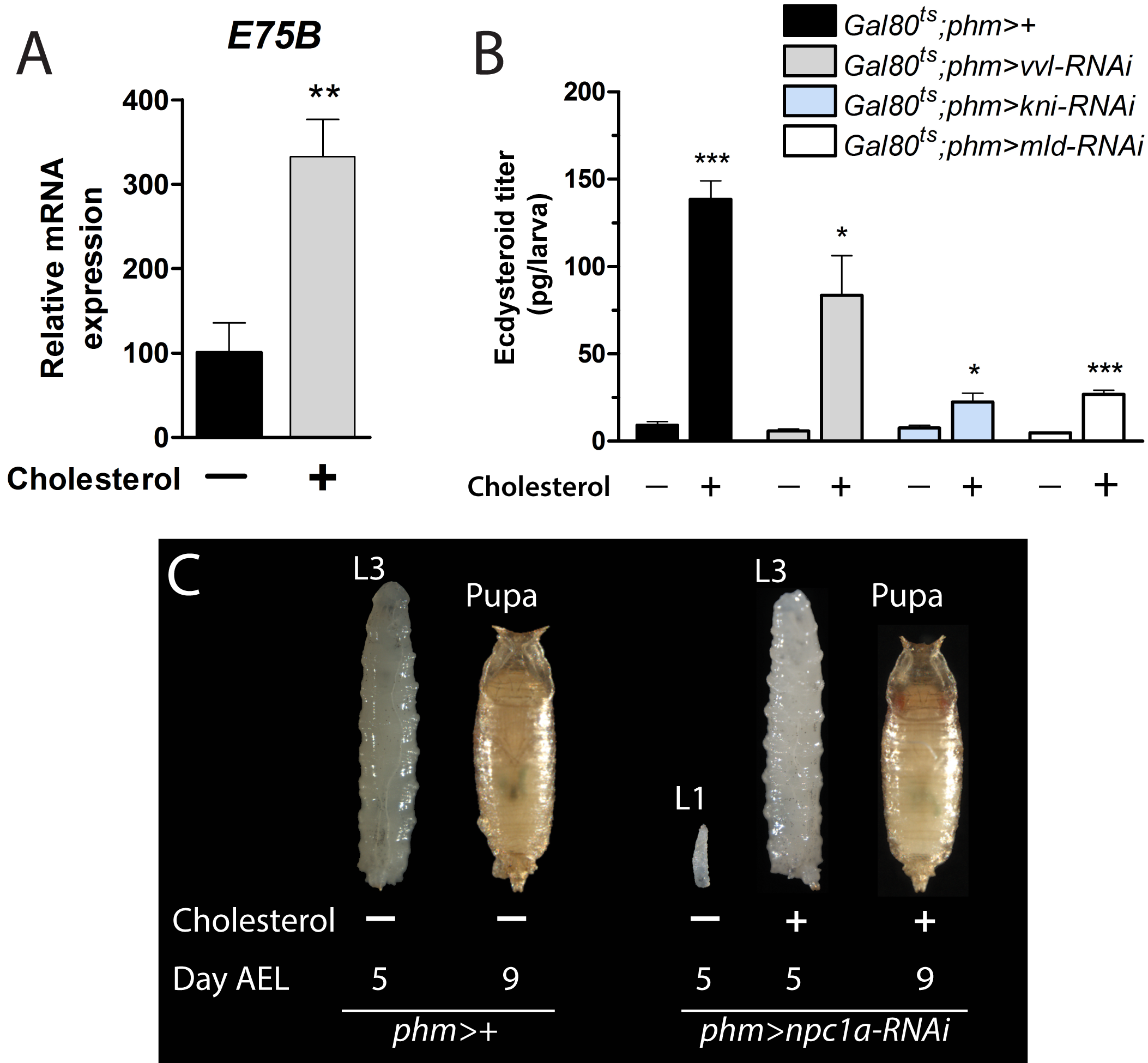

Supplement: Figure S3 — Effect of a high-cholesterol diet on ecdysteroid levels. (A) Effect of substrate concentrations on ecdysone biosynthesis was determined by measuring the transcription of E75B, as a proxy for ecdysone levels. Expression of E75B was determined in wild type (w1118) late L3 larvae 120 hours AEL, grown either on standard food (−) or a high-cholesterol diet (+). Elevated E75B expression in L3 larvae grown in the presence of cholesterol indicates that the amount of ecdysone produced depends on the supply of substrates (n = 4). **P<0.01, versus the control grown on standard food. (B) RNAi was induced in larvae 96 hours AEL by switching larvae from 18°C to 29°C and ecdysteroid levels were analyzed 36 hours later when control (Gal80ts;phm>+) larvae raised on high cholesterol exhibited wandering behavior, while animals raised on a standard diet were still in the pre-wandering stage. Ecdysteroid levels are increased in larvae raised on a high-cholesterol diet compared to standard food conditions, consistent with the accelerated development, indicated by the wandering behavior normally associated with the high-level ecdysone peak [24] (n = 4). *P<0.05, ***P<0.001, versus the Gal80ts;phm>+ control. (C) The PG must take up cholesterol from circulation to support ecdysone synthesis, a process that requires the function of Npc1a [26]. phm>npc1a-RNAi animals, with impaired delivery of cholesterol for ecdysone biosynthesis, arrest development in L1 when grown on a standard diet, but develop normally when cholesterol is increased by dietary supplementation. Error bars indicate s.e.m. (TIF) [file pgen.1004343.s003.tif]

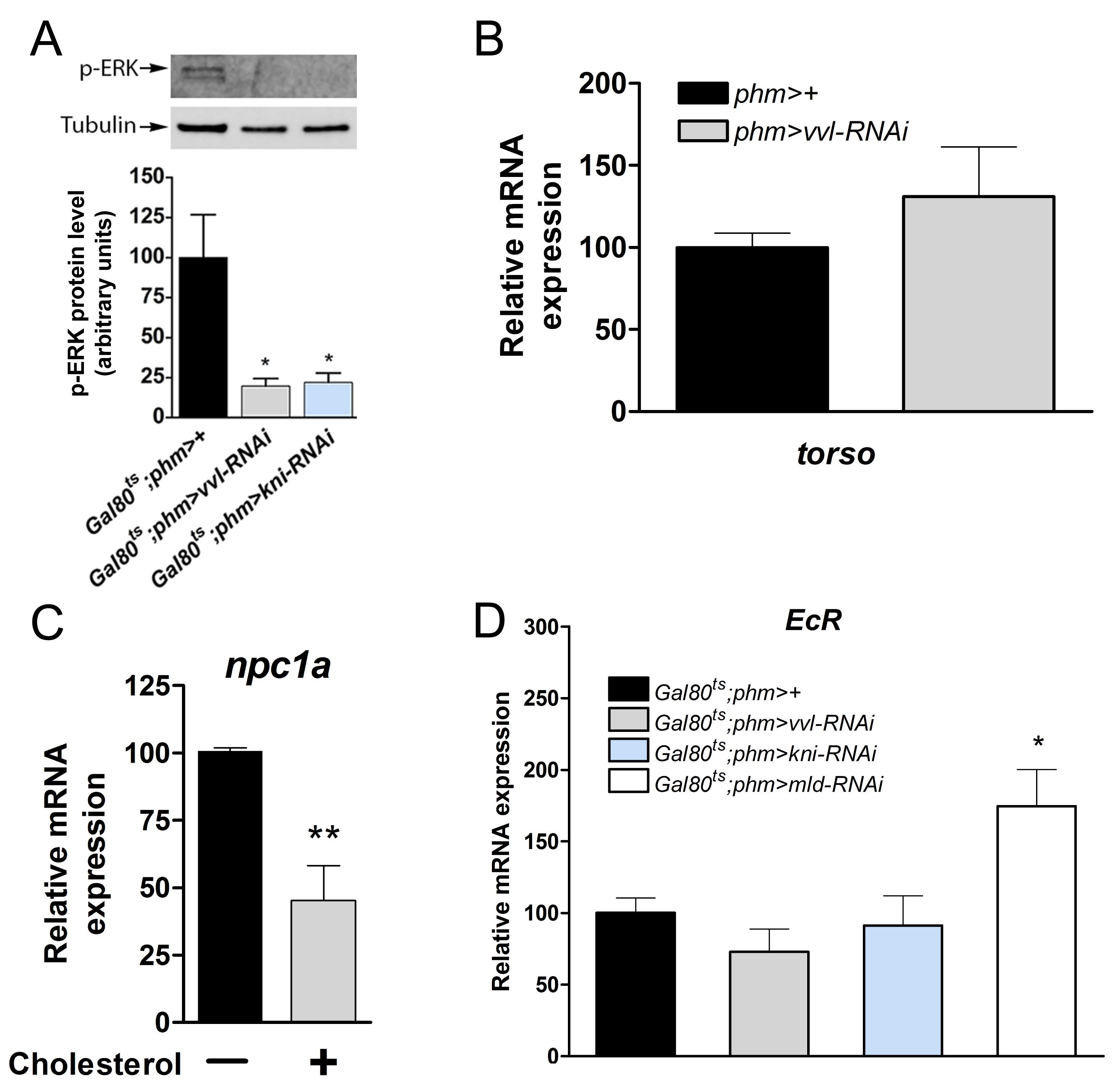

Supplement: Figure S4 — Effect of loss of vvl and kni on PTTH and ecdysone signaling and cholesterol feedback regulation of npc1a. (A) Quantification of phosphorylated ERK (p-ERK) levels in brain-ring gland complexes (BRGCs) from L3 animals determined by immunoblotting with an antibody specific for p-ERK. RNAi was induced 96 hours AEL by switching larvae from 18°C to 29°C and p-ERK levels were measured in BRGCs two days later. p-ERK levels were normalized to Tubulin. *P<0.01, versus the Gal80ts;phm>+ control. (B) Expression of torso was analyzed 36 hours AEL in mid-first instar larvae. (C) mRNA levels of npc1a were measured in wild type (w1118) L3 larvae 120 hours AEL, grown either on standard food (−) or on a high-cholesterol diet (+). Expression of npc1a was repressed by cholesterol (n = 4). **P<0.01, versus the control grown on standard food. (D) Expression of EcR in ring glands from L3 larvae two days after temperature induced activation of vvl-RNAi, kni-RNAi or mld-RNAi in the PG by switching larvae 96 hours AEL from 18°C to 29°C (n = 5). *P<0.01, versus the Gal80ts;phm>+ control. Error bars indicate s.e.m. (TIF) [file pgen.1004343.s004.tif]
