# Supplementary material for: Transcriptional Control of Steroid Biosynthesis Genes in the Drosophila Prothoracic Gland by Ventral Veins Lacking and Knirps
Source: PLoS Genet. 2014 Jun 19;10(6):e1004343. doi: 10.1371/journal.pgen.1004343 (PMC4063667; doi:10.1371/journal.pgen.1004343)
Supplement: Table S1 — Phenotypes with different RNAi lines using the strong phm> and weaker P0206> PG drivers. Crosses using the weak P0206> were raised at 29°C to enhance the activity of the Gal4/UAS system. Note that the vvl-RNAi #110723 and the kni-RNAi #34705 lines were used for all experiments unless otherwise stated. VDRC (Vienna Drosophila RNAi Center), BDSC (Bloomington Drosophila Stock Center). (DOCX) [file pgen.1004343.s005.docx]

| **RNAi target** | **Stock center and number** | **Phenotype** |
| --- | --- | --- |
| *phm>vvl-RNAi* | BDSC #26228 | L1 arrest |
| *P0206>vvl-RNAi* | BDSC #26228 | L3 arrest |
| *phm>vvl-RNAi* | VDRC #110723 | L1 arrest |
| *P0206>vvl-RNAi* | VDRC #110723 | Pupal lethal |
| *phm>kni-RNAi* | BDSC #34705 | L1/L2 arrest |
| *P0206>kni-RNAi* | BDSC #34705 | L2/L3 arrest |
| *phm>kni-RNAi* | VDRC #2980 | L3 arrest |
