# Supplementary material for: Transcriptional Control of Steroid Biosynthesis Genes in the Drosophila Prothoracic Gland by Ventral Veins Lacking and Knirps
Source: PLoS Genet. 2014 Jun 19;10(6):e1004343. doi: 10.1371/journal.pgen.1004343 (PMC4063667; doi:10.1371/journal.pgen.1004343)
Supplement: Table S2 — Development of vvl-RNAi, kni-RNAi and mld-RNAi larvae grown on a normal diet or on a high cholesterol diet. RNAi was induced 96 hours AEL by switching L2 larvae from 18°C to 29°C. (DOCX) [file pgen.1004343.s006.docx]

| **RNAi target** | **Phenotype** | |
| --- | --- | --- |
|  | **Normal diet** | **High cholesterol diet** |
| *Gal80^ts^;phm>vvl-RNAi* | L3 arrest | Pupal lethal |
| *Gal80^ts^;phm>kni-RNAi* | L3 arrest | L3 arrest |
| *Gal80^ts^;phm>mld-RNAi* | L3 arrest/Pupal lethal | No arrest (viable adults) |
