# Supplementary material for: Transcriptional Control of Steroid Biosynthesis Genes in the Drosophila Prothoracic Gland by Ventral Veins Lacking and Knirps
Source: PLoS Genet. 2014 Jun 19;10(6):e1004343. doi: 10.1371/journal.pgen.1004343 (PMC4063667; doi:10.1371/journal.pgen.1004343)
Supplement: Table S3 — List of oligos used for EMSA. Mutations introduced are underlined. (DOCX) [file pgen.1004343.s007.docx]

| **Oligo Name** | **Forward 5’-3’** | **Reverse 5’-3’** |
| --- | --- | --- |
| Unspecific Oligo | AACGTAGCTGATCGAATCGGTTAC | AGTAACCGATTCGATCAGCTACGT |
| phm_vvl wt | TGGGTGTAATGAATGTGCATACGA | ATCGTATGCACATTCATTACACCC |
| Phm_vvl_mut | TGGCATATGTGAATGTGCATACGA | ATCGTATGCACATTCACATATGCC |
| Vvl_wt | GCAGTCGACCATTTACATATTTACGTTTA | GTAAACGTAAATATGTAAATGGTCGACTG |
| Vvl_mut | GCAGTCGACCAGGGACCGATTTACGTTTA | GTAAACGTAAATCGGTCCCTGGTCGACTG |
| Phm_kni_wt | ACGGGCAATTTCATTTTCAAATT | AAATTTGAAAATGAAATTGCCCG |
| Phm_kni_mut | ACGGGCAATTTCCGTTTCAATTT | AAATTTGAAACGGAAATTGCCCG |
| Kni_wt | TGCATTAGAAAACTAGATCAG | CTGATCTAGTTTTCTAATGCA |
| Kni_mut | TGCATTAGCTAGCTAGATCAG | CTGATCTAGCTAGCTAATGCA |
