# Supplementary material for: Transcriptional Control of Steroid Biosynthesis Genes in the Drosophila Prothoracic Gland by Ventral Veins Lacking and Knirps
Source: PLoS Genet. 2014 Jun 19;10(6):e1004343. doi: 10.1371/journal.pgen.1004343 (PMC4063667; doi:10.1371/journal.pgen.1004343)
Supplement: Table S4 — List of primers used for qPCR. *Primers were adapted from [32]. (DOCX) [file pgen.1004343.s008.docx]

| **Gene** | **Forward 5´-3´** | **Reverse 5´-3´** |
| --- | --- | --- |
| *nvd | GGAAGCGTTGCTGACGACTGTG | TAAAGCCGTCCACTTCCTGCGA |
| *sro | AGCAGCTGAAGGTCGATAGC | GCGATTCGTGGCAGTAAAC |
| *spok | TATCTCTTGGGCACACTCGCTG | GCCGAGCTAAATTTCTCCGCTT |
| *phm | GGATTTCTTTCGGCGCGATGTG | TGCCTCAGTATCGAAAAGCCGT |
| *dib | TGCCCTCAATCCCTATCTGGTC | ACAGGGTCTTCACACCCATCTC |
| *sad | CCGCATTCAGCAGTCAGTGG | ACCTGCCGTGTACAAGGAGAG |
| vvl | CACTCCTGCATCCCTCAATAG | GGTGTATTCGGTGTGTGCTG |
| kni | GGAAGTGGAAAGAGATAGGGACA | TGTGAGTGTGTGTGTGGTGGT |
| mld | CTGGAGGTGGAGATGAACGA | ATCGGCTGGAGTGAGGAAC |
| tor | TGCTTGGATTGGTATCCCTATAA | TGGGTCACAGTAAGATTCTCTGG |
| InR | CTCAGCCATACCAGGGACTTT | CTCTCCATAACACCGCCATC |
| Npc1a | TTGCAACCAAGCAGTTAGCA | CAATTTTGAAGGGCTCTGGA |
| E75B | CAACAGCAACAACACCCAGA | CAGATCGGCACATGGCTTT |
| EcR | TGCGAAGAAGAGCAAGAAGG | CAGGTGAGGGCGTTGTAGTG |
| *Rpl23 | GACAACACCGGAGCCAAGAACC | GTTTGCGCTGCCGAATAACCAC. |
| RpL32 | TAAGCTGTCGCACAAATGGCG | AACGCGGTTCTGCATGAGCA |
